# ChatGPT vs. Gemini: could artificial intelligence safely improve the perioperative surgical flow while increasing patient awareness?

**DOI:** 10.1186/s40942-026-00923-1

**Published:** 2026-08-14

**Authors:** Maria Ludovica Ruggeri, Arianna Pennacchi, Alessandro Sferra, Matteo Fornaro, Alberto Quarta, Francesco Petti, Ruggero Tartaro, Corina Valentina de Santis Ciacci, Lisa Toto, Rodolfo Mastropasqua

**Affiliations:** 1 Eye Clinic, University Hospital of Chieti-Pescara, via dei Vestini 31, 66100 Chieti, Italy; 2 Department of Neuroscience, Imaging and Clinical Science, G. d’Annunzio University, Chieti, Italy; 3 Ophthalmology Clinic, Department of Medicine and Ageing Science, University “G. d’Annunzio” of Chieti-Pescara, Chieti, Italy

**Keywords:** Artificial intelligence, Innovation, Ophthalmology, Retinal detachment, Vitreoretinal surgery

## Abstract

**Aims:**

To evaluate the efficiency and safety of ChatGPT and Gemini in the surgical workflow, from patient admission to surgical planning.

**Methods:**

Twenty questions regarding preoperative and postoperative aspects of vitreoretinal surgery—most frequently asked by patients at our center—were submitted to ChatGPT (OpenAI) and Gemini (Google). Similarly, twenty surgical cases treated at our center were submitted to both platforms. The same questions and cases were evaluated by a panel of vitreoretinal surgeons with varying experience. Primary outcomes were overall reliability and inter-group agreement of the two chatbots; the secondary outcome was their ability to identify cases requiring ophthalmologist intervention.

**Results:**

In the clinical case dataset (*N* = 80), ChatGPT and Gemini performed comparably, with ChatGPT slightly outperforming Gemini in discussion timeliness (*p* = 0.019). In the patient inquiry dataset (*N* = 80), Gemini provided more complete, accurate, and timely clinical indications (*p* ≤ 0.011). Stratified analyses by surgeon experience (Junior vs. Senior) showed consistent evaluations across experience levels.

**Conclusions:**

ChatGPT and Gemini perform similarly in clinical case discussions, with ChatGPT slightly better in timeliness. For patient-oriented inquiries, Gemini delivers more accurate, complete, and up-to-date responses. While AI platforms demonstrate potential, their adoption in clinical practice should be preceded by thorough and rigorous evaluation.

**Supplementary Information:**

The online version contains supplementary material available at https://doi.org/10.1186/s40942-026-00923-1.

## Introduction

Beyond the surgical procedure itself, patient compliance represents one of the main factors strongly associated with the success of medical and surgical treatments. Recent studies in Ophthalmology have highlighted how adherence during the postoperative period significantly improves surgical outcomes [1–5]. Compliance, however, is influenced by multiple variables that must be carefully considered, including patient characteristics, the type and duration of medical or surgical therapy, and demographic and socioeconomic factors [6]. Specifically, younger age, African descent, shorter duration of medication use, lower educational level, and poorer scores on mental status and depression scales have been widely identified as factors influencing treatment adherence among patients with glaucoma [7–9]. Similarly, in corneal diseases, adherence to therapy appears to depend on environmental factors, treatment regimen complexity, patient-related variables, and physician-related factors. Interestingly, a recent study identified forgetfulness or falling asleep as the most common causes of poor adherence [10]. In the vitreoretinal setting, patient compliance plays a crucial role in perioperative management. Although postoperative inflammation control has been extensively investigated as a key component of postoperative care, insufficient patient adherence may negatively affect outcomes not only in terms of eye drops administration—a topic that has been relatively underexplored in ophthalmology—but also regarding postoperative behavior [11–13]. Indeed, patients are often required to maintain specific positioning according to the surgical procedure performed. This is particularly relevant in primary full-thickness macular holes (FTMHs), where several authors have emphasized the importance of the face-down position (FDP) after pars plana vitrectomy (PPV) to maximize surgical success [14]. Artificial intelligence (AI) represents one of the most significant technological advancements of the current era, profoundly impacting daily life as well as medical and surgical practice [15]. Due to their accessibility and productivity, large language model–based chatbots such as ChatGPT (OpenAI) and Gemini (Google) have achieved widespread diffusion and recognition, being increasingly used by both surgeons and patients. Recently, Al-Latayfeh et al. evaluated the accuracy of different artificial intelligence chatbots in responding to patient-based vitreoretinal questions, reporting high levels of accuracy and reproducibility comparable to clinical standards in both medical and surgical retinal scenarios [16]. Similarly, Carlà et al., investigating AI applications in retinal detachment surgical planning, found substantial agreement between expert surgeons, Google Gemini, and ChatGPT [17]. By simulating potential patient concerns before and after surgery and by analyzing different vitreoretinal surgical scenarios, AI chatbots may influence perioperative management and potentially impact operative decision-making. Furthermore, no studies have yet analyzed the limitations of such AI-assisted programs by testing situations in which the necessity of human intervention is acknowledged. The aim of the present study was to evaluate the performance of ChatGPT and Google Gemini in the operative setting by assessing their accuracy, precision, and reproducibility in surgical planning and perioperative patient management.

## Materials and methods

This study was approved by the Institutional Review Board (IRB) and was conducted at the Ophthalmology Clinic of SS. Annunziata Hospital, “G. d’Annunzio” University of Chieti–Pescara, Chieti, Italy, between February 1, 2025, and January 31, 2026. Two distinct domains were analyzed to assess the reliability of ChatGPT (OpenAI) and Gemini (Google) within the perioperative workflow: the patient perspective and the surgeon perspective.

The analysis was performed using the free versions of ChatGPT (GPT-4o, OpenAI) and Gemini (Gemini 2.5 Pro, Google), reflecting the versions available to the general public at the time of data collection.

**Fig. 1:**
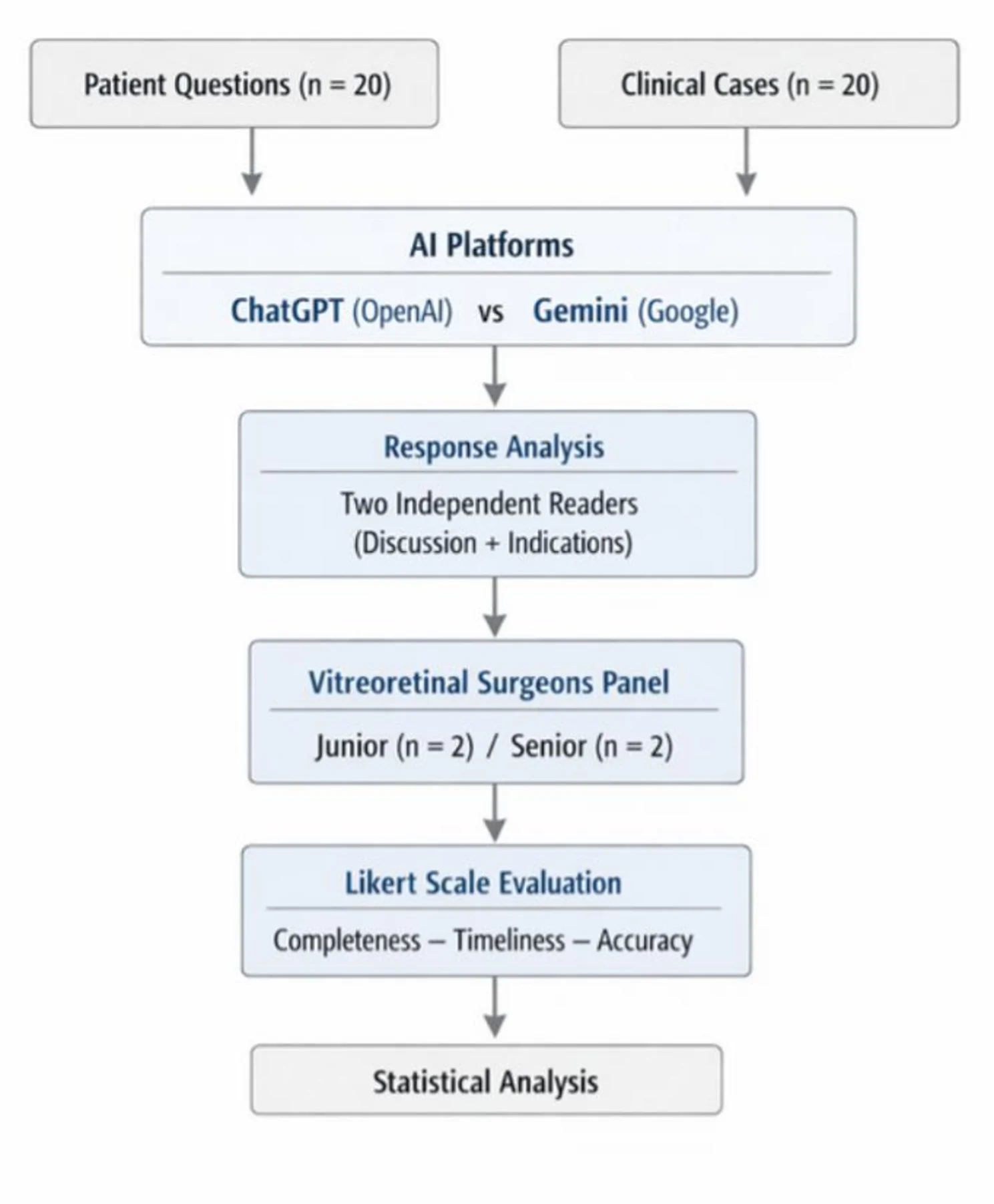
Study design and evaluation workflow. Workflow representation of the study methodology. A total of 40 questions were submitted to ChatGPT and Gemini (*n* = 20 patient-oriented questions and *n* = 20 clinical cases. Responses were independently analyzed by two readers, focusing on discussion and clinical indications. Subsequently, a panel of vitreoretinal surgeons (junior, *n* = 2; senior, *n* = 2) evaluated the outputs using a Likert scale assessing completeness, timeliness, and accuracy

A panel of vitreoretinal surgeons with different levels of experience was established. Among them, two were classified as junior surgeons (with < 10 years of surgical experience), and two were classified as senior surgeons (fellowship-trained vitreoretinal specialists with > 10 years of experience). The primary outcomes were the overall reliability and inter-group reliability of the two chatbots. The secondary outcome was the ability of ChatGPT and Gemini to recognize cases requiring ophthalmologist intervention. Figure 1 shows the study methodology flowchart.

### The patient perspective

A total of 20 questions regarding preoperative and postoperative aspects of vitreoretinal surgery, most frequently asked by patients undergoing surgery at our center during the enrollment period, were submitted to the two widely used artificial intelligence platforms, ChatGPT (OpenAI) and Gemini (Google) (Suppl. Table 1).

All questions were entered into both platforms using the same standardized input format. Two independent readers (AS and MF) analyzed the chatbot-generated responses and structured each reply into two predefined sections: discussion and indications.

Each vitreoretinal surgeon independently evaluated both sections according to three predefined parameters: completeness, up-to-dateness, and accuracy. Each item was graded using a 5-point Likert scale ranging from 0 to 4 (0 = strongly disagree; 1 = disagree; 2 = partially agree; 3 = agree; 4 = strongly agree). The scores were collected and analyzed both overall and according to surgeon experience level.

### The vitreoretinal surgeons’ perspective

A total of 20 clinical cases of vitreoretinal surgery treated at our center during the enrollment period were submitted to ChatGPT and Gemini.

The clinical scenarios included cases of vitreous hemorrhage, macular interface disorders, and retinal detachment. Specifically, the cases comprised surgical management of vitreous hemorrhage (*n* = 5), macular interface diseases (*n* = 7), and rhegmatogenous retinal detachment (*n* = 6), ocular trauma (*n* = 4) (Suppl. Table 2).

For each case, the platforms generated a structured response, which was independently reviewed by two readers (AS and MF) and divided into *discussion* and *indications* sections. These sections were subsequently evaluated by the predefined panel of vitreoretinal surgeons using the same 5-point Likert scale and according to the same assessment domains (completeness, up-to-dateness, and accuracy).

### Statistical analysis

Statistical analysis was performed to compare the reliability of the two platforms under investigation. Participants were allocated into two groups according to the platform used, and comparisons were conducted both in terms of overall performance and after stratification by surgeon experience level (junior vs. senior). Continuous variables were reported as mean and standard deviation (SD) or median and interquartile range (IQR), as appropriate, based on data distribution assessed using normality tests. Between-group differences were evaluated using the independent-samples t test for normally distributed variables or the Mann–Whitney U test for non-normally distributed variables. Categorical variables were expressed as frequencies and percentages and compared using the chi-square test or Fisher’s exact test, as appropriate. To further investigate the potential interaction between platform type and surgeon experience level on reliability outcomes, a two-way analysis was conducted using a general linear model, including main effects and interaction terms. When appropriate, effect sizes and 95% confidence intervals (CIs) were calculated to provide an estimate of the magnitude and precision of the observed differences. A two-tailed p value < 0.05 was considered statistically significant. All statistical analyses were performed using IBM SPSS Statistics (IBM Corp., Armonk, NY, USA).

## Results

**Fig. 2:**
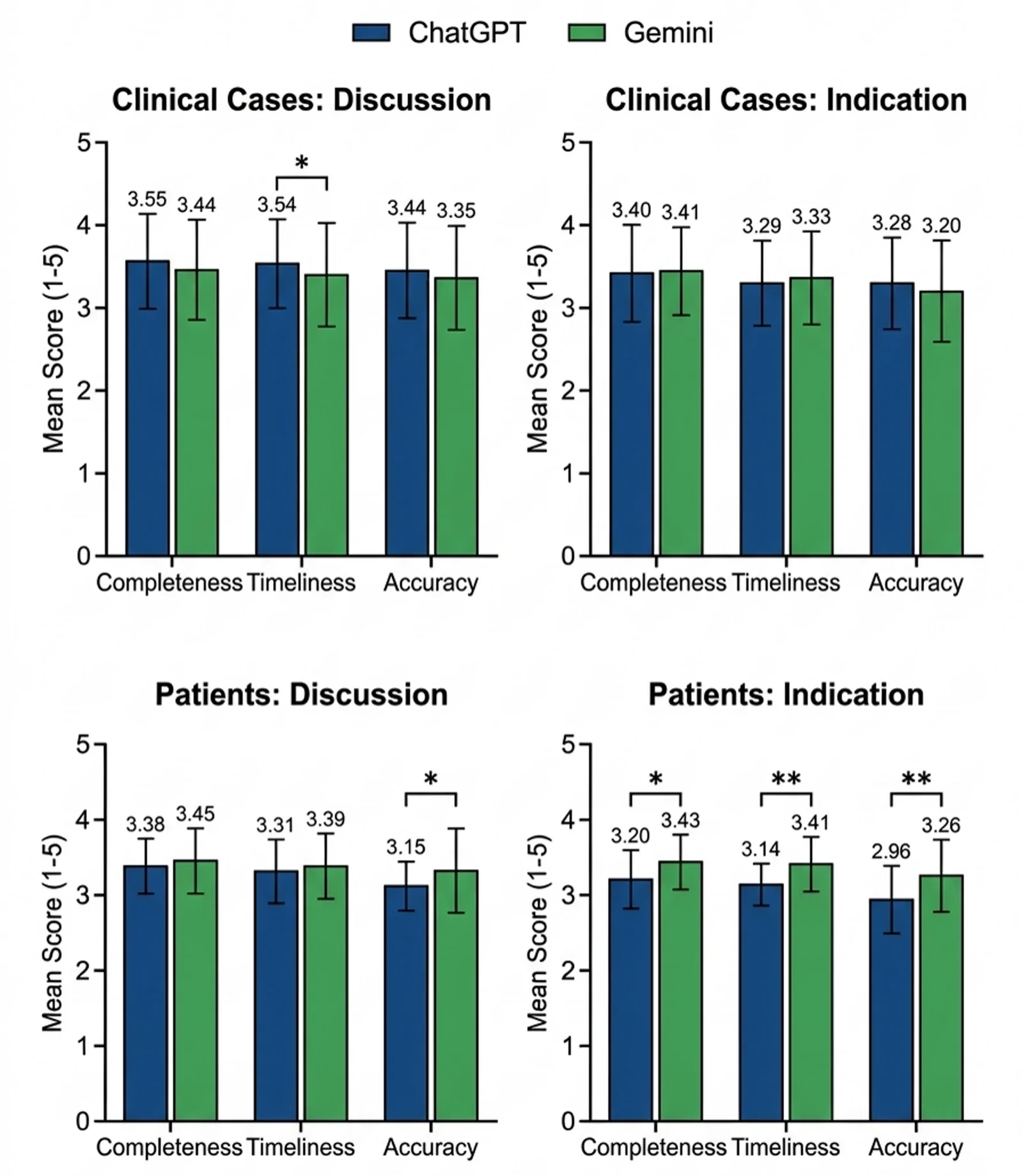
Comparative performance of ChatGPT and Gemini across clinical scenarios. Bar graphs illustrating mean Likert scores (± SD) for completeness, timeliness, and accuracy assigned to responses generated by ChatGPT and Gemini in two settings: clinical cases and patient-oriented questions. In clinical cases, both platforms demonstrated comparable performance, with a statistically significant advantage for ChatGPT in discussion timeliness (*p* < 0.05). No significant differences were observed in the indication domain. In patient-oriented queries, Gemini achieved higher scores overall, with a statistically significant advantage in discussion accuracy (*p* < 0.05) and across all indication metrics, including completeness (*p* < 0.05), timeliness (*p* < 0.01), and accuracy (*p* < 0.01). Asterisks indicate statistically significant differences between platforms (*p* < 0.05; *p < 0.01)

In the “Clinical Cases” dataset (*N* = 80 evaluations), the two platforms demonstrated largely comparable performance across all assessed metrics (Fig. 2). In the Discussion domain, no statistically significant differences were observed for Completeness (3.55 ± 0.61 vs. 3.44 ± 0.63; *p* = 0.072) or Accuracy (3.44 ± 0.65 vs. 3.35 ± 0.68; *p* = 0.124), whereas ChatGPT showed a modest but statistically significant advantage in Timeliness (3.54 ± 0.64 vs. 3.38 ± 0.68; *p* = 0.019) (Fig. 2). In the Indication domain, no significant differences were identified between platforms for Completeness (*p* = 0.866), Timeliness (*p* = 0.663), or Accuracy (*p* = 0.310). No significant interaction between platform and surgeon experience was observed (*p* > 0.05 for all metrics), indicating consistent evaluations across experience levels. Stratified analysis revealed a more pronounced non-significant, difference in Discussion Timeliness among senior surgeons (*p* = 0.07) compared to junior surgeons (*p* = 0.13), with a similar directional trend. In contrast, in the “Patients” dataset (*N* = 80 evaluations), Gemini demonstrated superior performance, particularly in the Indication domain. Within the Discussion domain, differences were not statistically significant for Completeness (3.38 ± 0.58 vs. 3.45 ± 0.53; *p* = 0.180) or Timeliness (3.31 ± 0.61 vs. 3.39 ± 0.52; *p* = 0.221), although Gemini achieved significantly higher Accuracy scores (3.33 ± 0.63 vs. 3.15 ± 0.64; *p* = 0.025). In the Indication domain, Gemini significantly outperformed ChatGPT across all metrics, including Completeness (3.43 ± 0.57 vs. 3.20 ± 0.82; *p* = 0.011), Timeliness (3.41 ± 0.54 vs. 3.14 ± 0.69; *p* = 0.001), and Accuracy (3.26 ± 0.69 vs. 2.96 ± 0.77; *p* = 0.002). No significant interaction between platform and surgeon experience was detected (*p* > 0.05), confirming that these findings were consistent across both junior and senior evaluators. Stratified analysis showed that junior surgeons rated Gemini significantly higher across all Indication metrics, whereas senior surgeons confirmed its superiority in Timeliness (*p* = 0.018) and Accuracy (*p* = 0.037), without reaching statistical significance for Completeness (*p* = 0.15). Finally, in patient-related queries, Gemini more frequently recommended contacting the treating surgeon compared to ChatGPT (*n* = 7 vs. *n* = 7), while in clinical case scenarios, Gemini suggested referral to an ophthalmologist more often (*n* = 1 vs. *n* = 0).

## Discussion

The widespread adoption of artificial intelligence (AI)-based platforms in medicine has progressively reshaped teaching, training, and, potentially, the clinical–diagnostic process [18]. The need for continuous updating represents a cornerstone of modern medicine, particularly in ophthalmology, where novel methodologies and therapeutic approaches—such as gene therapy—are transforming the management of conditions historically considered untreatable [19]. In this evolving landscape, AI is increasingly influencing both medical education and clinical reasoning [20]. Several studies have investigated the impact of AI on medical education. A recent review by Ren et al. demonstrated that, when properly integrated, AI can enhance personalized learning while fostering a more inclusive and ethically grounded educational framework, highlighting its significant pedagogical potential and ethical implications [21]. In line with this perspective, our group previously explored the role of mixed reality in vitreoretinal education, proposing an innovative teaching model associated with high levels of safety and user satisfaction [22]. Notably, the same technology had already been implemented in the surgical setting, demonstrating feasibility while preserving surgical safety and sterility [23]. 

In this context, AI chatbots are becoming increasingly integrated into patients’ daily lives and may therefore serve as valuable tools to enhance patient awareness and education throughout the perioperative period. Our analysis highlights that these platforms are capable of providing information on perioperative recommendations; however, their use still requires the involvement of healthcare professionals who can interpret the generated responses and address any remaining patient questions or concerns. Consequently, AI chatbots should be regarded as complementary to, rather than replacements for, the patient–physician relationship. Accordingly, the patient–ophthalmologist alliance may be strengthened by incorporating AI tools as adjunctive educational resources within the perioperative care pathway.

Similarly, AI-based models have increasingly contributed to diagnostic processes. In high-prevalence conditions such as diabetic retinopathy and age-related macular degeneration, AI systems have enabled early identification of disease biomarkers, thereby streamlining diagnostic workflows and facilitating large-scale screening programs [24, 25]. These approaches have also been extended to vitreoretinal diseases, as reported by Mariotti et al. who analyzed biomarkers associated with epiretinal membranes before and after surgical intervention, further supporting the role of AI in disease characterization [26]. As AI applications continue to evolve, their integration is progressively extending toward the surgical domain, where safety and efficiency are paramount. In this context, Al-Latayfeh et al. evaluated the accuracy and reproducibility of generative AI chatbots, reporting high performance and proposing their use as supportive tools for patient education in vitreoretinal care [16]. Conversely, Carlà et al. investigated the capability of AI systems to assist in surgical planning, comparing ChatGPT and Google Gemini in the management of retinal detachment cases [17]. Within this framework, the present study provides a comparative analysis of two of the most widely used AI platforms, ChatGPT and Gemini, across two key domains relevant to vitreoretinal surgery: structured clinical case discussion and patient-oriented queries. Our findings revealed a clear dichotomy between these scenarios. In structured clinical case discussions, both platforms demonstrated comparable performance, with a slight advantage for ChatGPT in terms of timeliness. In contrast, in patient-oriented queries, Gemini provided significantly more accurate, complete, and timely responses, particularly with regard to clinical indications. These findings suggest that both platforms perform reliably in structured contexts, whereas Gemini may exhibit superior capability in handling less structured and more generalizable queries, such as those arising in patient communication. Importantly, surgeon experience did not significantly influence platform evaluation, as no interaction effects were observed between experience level and performance outcomes. This finding supports the robustness and generalizability of the results across different levels of clinical expertise. An additional noteworthy observation is that Gemini more frequently recommended consultation with an ophthalmologist, potentially reflecting a higher degree of clinical caution and an implicit recognition of the limitations of AI systems. This behavior may represent an important safety feature, particularly in patient-facing applications. However, our analysis has limitation. First the Likert scale implies subjectivity. Secondly, the simulated setting, despite the use of real case scenario, may have caused a question selection bias.

Furthermore, we must acknowledge that chatbot performance may vary according to the specific model and version used. The reported evaluation was based exclusively on text, since no clinical images were provided to the chatbots.

## Conclusions

While the rapid advancement of AI technologies should be encouraged, their integration into clinical practice must be approached with caution to ensure that they enhance, rather than compromise, clinical decision-making. Overall, AI systems appear effective in supporting the clinical–diagnostic process, including applications extending toward the operating room, provided they are used as adjunctive tools under appropriate clinical supervision. In particular, these models may be valuable in patient education and triage, while ChatGPT may offer additional benefits in facilitating structured clinical reasoning and surgical planning. Nevertheless, all AI-generated outputs should be critically appraised by clinicians, ensuring that technological innovation supports, rather than replaces, medical expertise.

## Supplementary Information

Below is the link to the electronic supplementary material.

Supplementary Material 1

Supplementary Material 2

## Data Availability

No datasets were generated or analysed during the current study.
