# Supplementary material for: ChatGPT vs. Gemini: could artificial intelligence safely improve the perioperative surgical flow while increasing patient awareness?
Source: Int J Retina Vitreous. 2026 Aug 14;12:152. doi: 10.1186/s40942-026-00923-1 (PMC13756931; doi:10.1186/s40942-026-00923-1)
Supplement: Supplementary file 1 [file 40942_2026_923_MOESM1_ESM.docx]

**Supplementary Table 1. *The Patient Perspective***

After vitrectomy surgery for retinal detachment treated with gas tamponade, for how many hours and how many days must I maintain the position indicated by the surgeon?

At my follow-up visit the day after vitrectomy surgery for retinal detachment treated with gas tamponade, I was told I have low intraocular pressure. Why is that? Is this normal?

After vitrectomy surgery for retinal detachment treated with silicone oil as a tamponade, why is my vision blurry and changes throughout the day?

After retinal detachment surgery, how long does it take to return to normal daily life?

After 30 days of surgery for an epiretinal membrane, my vision is still a little distorted, just like before surgery. Is this normal?

Why did I also undergo cataract surgery during retinal detachment surgery?

After vitrectomy surgery for retinal detachment, is it normal to still see some flashes of light?

After surgery for a full-thickness macular hole, is it normal to still see a central black spot?

Why did I have to start using eye drops to lower my intraocular pressure after vitrectomy surgery for retinal detachment?

After vitrectomy surgery, I see small moving spots or “floaters” that were not present before the operation. Should I be concerned?

After a vitrectomy with silicone oil tamponade for retinal detachment, how long after should I have a second procedure to remove the oil?

Can glaucoma eye drops be used after silicone oil vitrectomy?

During the epiretinal membrane surgery, I feel like I moved my eye. Three days after the surgery, I still can't see well. Could I have damaged something?

Can I resume anticoagulants after vitrectomy for vitreous hemorrhage?

One day after retinal detachment surgery, can I watch television and light?

After retinal detachment surgery, I coughed repeatedly on my way home. Could this have compromised the surgery?

After vitrectomy surgery, I notice that my operated eye is more sensitive to light and becomes tired when reading or using digital devices. Is this normal during recovery?

The day after vitrectomy surgery for retinal detachment, I lifted 20 kg. I was told not to do any physical exertion. Could this have compromised the surgery?

I had vitreoretinal surgery and didn't start the eye drops they recommended on the same day. Could I have compromised something?

After vitrectomy surgery with gas tamponade, I still see a moving shadow or a “bubble” in my vision several days after surgery. Is this normal, and how long will it take to disappear?

Overview of patient-derived questions regarding preoperative and postoperative aspects of vitreoretinal surgery. Twenty questions were selected based on the most frequent concerns expressed by patients undergoing surgery at our institution during the enrollment period.
