# Supplementary material for: ChatGPT vs. Gemini: could artificial intelligence safely improve the perioperative surgical flow while increasing patient awareness?
Source: Int J Retina Vitreous. 2026 Aug 14;12:152. doi: 10.1186/s40942-026-00923-1 (PMC13756931; doi:10.1186/s40942-026-00923-1)
Supplement: Supplementary file 2 [file 40942_2026_923_MOESM2_ESM.docx]

### **Supplementary Table 2. *The Vitreoretinal Surgeons’ Perspective***

A 48-year-old male patient with macular retinal detachment and a single superotemporal retinal tear in a phakic patient (initial yellowing of the lens) with high myopia (axial length 30 mm). What do you recommend?

Bullous inferior retinal detachment with macula off and small retinal hole at 12 o'clock (Lincoff IV), 70-year-old male patient with phakic vision, 1/10. What do you recommend I do?

Retinal detachment macula off vision 1/30 with symptoms onset 1 week ago, pseudophakic patient with hydrophilic acrylic IOL with multiple retinal breaks at 2, 3 and 7 o'clock. What do you recommend I do?

A highly myopic aphakic patient with subtotal retinal detachment with type C PVR originating from a full-thickness macular hole. What do you recommend?

Retinal detachment in pseudophakic eye with multiple inferior macula tears. Symptoms began 15 days ago with decreased vision. Current vision is 1/10. A specialist history of penetrating keratotomy for post-infectious central corneal leukoma (currently a transparent flap) and subsequent onset of post-PK glaucoma treated with a Paul glaucoma implant in the ciliary sulcus. What do you recommend I do?

Patient with retinal detachment at the 10-12 o'clock macula with a tear at the 11 o'clock macula treated with pneumatic retinopexy (SF6) and cryotherapy of the tear. Three days after surgery, a new retinal tear was detected in the inferior 5 o'clock macula. What do you recommend?

Patient with hemorrhagic PDV, impaired hand and fundus motion vision due to vitreous hemorrhage, symptoms onset 1 day earlier. B-scan ultrasound confirms vitreous hemorrhage and presence of a frank superotemporal retinal tear. Phakic patient, 55 years old. What do you recommend?

Patient with proliferative diabetic retinopathy (PDR), counting fingers vision due to dense vitreous hemorrhage, symptoms onset 5 days earlier. B-scan ultrasound shows attached retina with no retinal tears or tractional retinal detachment. Pseudophakic patient, 68 years old, on aspirin therapy. **What do you recommend?**

Patient with acute posterior vitreous detachment and dense vitreous hemorrhage, visual acuity reduced to hand motion, symptoms onset 12 hours earlier. B-scan ultrasound is inconclusive because of dense hemorrhage and does not clearly identify retinal tears or retinal detachment. Phakic patient, 61 years old, high myope (-9.00 D). **What do you recommend?**

Patient with branch retinal vein occlusion previously treated with anti-VEGF injections, presenting with recurrent vitreous hemorrhage and best-corrected visual acuity of counting fingers. Symptoms started 2 weeks earlier. B-scan ultrasound shows attached retina without retinal breaks. Pseudophakic patient, 72 years old, anticoagulated with apixaban. **What do you recommend?**

Patient with retinal tear treated with laser photocoagulation 3 days ago, now presenting with persistent dense vitreous hemorrhage and vision reduced to counting fingers. B-scan ultrasound shows an attached retina with no progression to retinal detachment. Phakic patient, 58 years old. **What do you recommend?**

A lamellar hole with a residual thickness of 90 microns has been present in a patient who has been complaining of metamorphopsia for 6 months, which is interfering with his work as a musician. What do you recommend I do?

A 75-year-old pseudophakic patient with stage IV epiretinal membrane (according to Govetto), metamorphopsia, and 2/10 vision. What do you recommend?

Patient with an idiopathic epiretinal membrane, best-corrected visual acuity of 20/25, significant metamorphopsia for 8 months affecting daily reading and computer work. OCT shows marked retinal wrinkling and central macular thickness of 510 µm. Phakic patient, 63 years old. **What do you recommend?**

Patient with vitreomacular traction syndrome, best-corrected visual acuity of 20/40, progressive visual distortion over the last 4 months. OCT demonstrates focal vitreomacular adhesion with foveal deformation but no full-thickness macular hole. Phakic patient, 59 years old. **What do you recommend?**

Patient with a full-thickness macular hole measuring 320 µm (minimum linear diameter), symptoms onset 3 weeks earlier, best-corrected visual acuity of 20/100. Phakic patient, 66 years old. **What do you recommend?**

Patient with an epiretinal membrane associated with a lamellar macular hole. Best-corrected visual acuity is 20/40, but the patient reports progressive metamorphopsia and impaired depth perception for the past year. OCT shows ellipsoid zone preservation and residual foveal thickness of 110 µm. Pseudophakic patient, 71 years old. **What do you recommend?**

Patient with myopic foveoschisis (axial length 30.5 mm), best-corrected visual acuity of 20/80, progressive visual decline over 9 months. OCT shows foveoschisis with focal foveal detachment but no full-thickness macular hole. Phakic patient, 58 years old. **What do you recommend?**

A 70-year-old patient suffered an ocular trauma 4 years ago, followed by a central scotoma. A FTMH was diagnosed. He was treated with vitrectomy and an inverted-flap technique. He has never recovered vision above 1/20. Three years later, a FTMH recurs, with initial elevation of the edges of the hole and subfoveal atrophy. What do you recommend?

A 35-year-old patient with post-traumatic temporal retinal dialysis. What do you recommend?

A 70-year-old patient with pseudoexfoliative syndrome who underwent scleral-fixated intraocular lens implantation presented with a lens fall into the vitreous cavity following trauma, resulting in a retinal tear one papillary diameter away from the inferotemporal vascular arch. What do you recommend?

Patient with blunt ocular trauma 2 months earlier, presenting with persistent visual distortion and reduced visual acuity (20/80). OCT shows a traumatic macular hole of 300 µm with partial spontaneous closure signs and persistent vitreomacular adhesion. The patient is highly symptomatic and works as a professional photographer. Phakic patient, 35 years old. **What do you recommend?**

**Supplementary table 2.** Overview of the clinical scenarios presented. The survey included cases involving vitreous hemorrhage (n = 5), macular interface disorders (n = 7), rhegmatogenous retinal detachment (n = 6), and ocular trauma (n = 4), with a focus on decision-making surgical management.
